# Inter-cluster separation induced change in charge transport mechanism in Ni_40_Pd_60_ nanoclusters

**DOI:** 10.1038/s41598-019-43581-0

**Published:** 2019-05-17

**Authors:** S. G. Praveen, C. Bansal, D. Jaiswal Nagar

**Affiliations:** 1 School of Physics, Indian Institute of Science Education and Research Thiruvanthapuram, Vithura, Thiruvananthapuram, 695551 India; 2 School of Physics, University of Hyderabad, Hyderabad, 500046 India

**Keywords:** Nanoscale materials, Nanoscale materials

## Abstract

Nanoclusters offer a fascinating possibility of studying the evolution of properties of a physical system by varying the number, size and inter-cluster separation of a given cluster to go from one limit to another. By systematically varying the inter-cluster separation in a nanocluster assembly of Ni_40_Pd_60_ alloy, that is known to be a metal in bulk, we observe an unusual and hitherto unreported, spatial dimension change as well as a change in the transport mechanism. In the nanocluster form, the temperature dependent resistance shows an activated behavior for virtually all inter-cluster separations, contrary to, the bulk metallic behaviour. At large average inter-cluster separation, the transport happens via three dimensional Efros-Shklovskii hopping, due to the opening of a Coulomb gap at the Fermi surface. With a reduction in the inter-cluster separation, the transport mechanism changes from three dimensional Efros-Shklovskii hopping to that of a three dimensional Mott variable range hopping (VRH) due to the closing up of the gap. With a further reduction in average inter-cluster separation, the three dimensional Mott VRH changes to that of a two dimensional Mott VRH with additional signatures of an insulator to a weak metal-like transition in this particular assembly. So, nanoclusters offer a paradigm for studying the important problem of evolution of charge transport in physical systems with the possibility of directly tuning the average inter-cluster separation enabling the system to go from insulating to metallic limit via intermediate changes in the charge transport mechanism.

## Introduction

Transport of a degenerate electron gas in a disordered environment has been a subject of intense study^1–6^. For instance, Anderson^1^ showed that at low concentration of impurities, transport happens via quantum mechanical jumps of the mobile entities (electrons or spins) from site to site, namely, traps in a conduction band and if the traps have a continuous range of energies, then the disordered lattice has no diffusion. Abrahams *et al*.^2^ and Lee *et al*.^3^, showed that two dimensions is the lower limit for disordered transport where the conductivity has a steep cross-over from an exponential (in the limit of strong localisation) to that of logarithmic (in the case of weak localisation). Here, even a non-interacting electron gas will be localized in the presence of an arbitrary small disorder and there would be no metallic conduction. However, several experiments on two dimensional systems in Silicon have suggested otherwise^7,8^. In three dimensions, however, there is no restriction on the electron transport and it was first suggested by Mott^4–6^ that the low temperature transport happens via thermally activated hopping between localized states around the Fermi level E_*F*_, the phenomenon being called variable range hopping (VRH) or Mott variable range hopping. The temperature dependence of resistance for Mott-VRH in three dimension is given by:

1 $$R(T)={R}_{0}{\exp }{({T}_{0}/T)}^{(1/4)}$$

where R_0_ is the zero temperature resistance and T_0_, a characteristic temperature. In general, for a D dimensional transport, the above equation is replaced as^4–6^:

2 $$R(T)={R}_{0}{\exp }{({T}_{0}/T)}^{p}$$

where

3 $$p=\frac{1}{D+1}$$

p is called the hopping exponent So, for a Mott-VRH in three dimensions, p = 1/4. In deriving Eq. (1), Mott assumed that the density of states near E_*F*_ is either constant or varies slowly as a function of energy. However, Pollok^9^ showed that if the Fermi energy lies in the range of energies where the states are localised, then a Coulomb gap opens at the density of states due to Coulomb interaction between the localized states. This led Efros and Shklovskii^10,11^ (E-S) to consider a density of states that vary linearly with energy near E_*F*_, resulting in a change in the value of the hopping exponent p from 1/4 to 1/2, irrespective of the dimensions. The varying kind of transport mechanism have been observed, primarily, in different semiconducting systems, namely, Si^7^, InP^12,13^, CdSe^14,15^, GaAs^16^, GaAs/Al_0.3_Ga_0.7_As heterostructures^17^, GaAl_0.3_Ga_0.7_As:Si^18^, graphene oxide sheets^19^ etc. but also in amorphous indium oxide In_*x*_O_*y*_ films^20^ as well as metallic Bi films^21^, Fe_50_X_50_ (X = Au, Pt and Pd)^22^ and Ag^23^ nanoclusters etc. Although each type of hopping transport has been observed in many systems, an observation of a change in the hopping mechanism from E-S kind to Mott VRH has been fewer and, mostly, as a function of temperature^14,15,18,20^. There have been much lesser observations of both type of conduction mechanisms in the same material as a function of either concentration^7,16^ or magnetic field^12,17^.

Apart from the hopping mechanism of charge transport that happens at low enough temperatures via electrons transiently localised at sites, the other well-known mechanism of charge transport is via quantum-mechanical tunnelling^24–27^ which is expected to become important for electrical conductivity if large regions of a highly conductive (’metallic’) phase are separated from each other by an insulating phase^24–27^. Such a scenario is realised in granular metal films^24,28–32^ which are either composite materials consisting of metals and insulators, for ex. Ni-SiO_2_ (made by mixing metallic Si with insulating SiO_2_)^30,31^, or metal island films made during the initial phases of a thin metal film deposition^32^. Due to an exponential dependence of the tunneling probability on the width of the insulating layer, it is expected that the tunneling mechanism would be dominant only at very small widths of the insulating layer. At low widths, when both the mechanism can contribute to charge transport, it is often difficult to distinguish one from the other. Motts mechanism of conduction is assumed to arise due to a sequential or percolative competition between space and energy^33^. However, the competition between space and energy can also arise due to the different number of paths a particle can take to cross a Coulomb barrier of a given height. In this case, tunnelling mechanisms would lead to a T^−1^ variation of resistivity^32^. From an energy dependent contribution to entropy calculations of transmission probability of a Coulomb barrier, Anglada *et al*.^34^ found that in the limit of thin insulating barrier thickness, the dominant mode of charge transport would be via Motts variable range hopping, if the hopping exponent p equals 1/3.

It would be very instructive if the various kinds of charge transport in a given system could be studied systematically by varying only a single parameter. Nanoclusters offer such a fascinating possibility and are a class of materials that lie in the intermediate range of atoms and bulk with properties that are neither microscopic nor macroscopic. The study of these systems is important both from a technological point of view since they not only help us in understanding problems related to micro-electronic industry, catalysis, aerosols, chemisorption etc. but also to understand fundamental physics like quantum confinement, Andersons criteria in superconductivity, interplay of various energy scales etc. from a bottom-up approach^22,23,35–40^. Nanoclusters can be conveniently produced using a nano-cluster deposition system where the size of a given nanocluster, the number of atoms within a nanocluster as well as the inter-cluster separation can be controlled by controlling the various parameters of the deposition^23,35,36,41,42^. These nano-cluster assembled films differ significantly from previously studied metal films, which consist of crystallite grains separated by grain boundaries^30,31^. In a nanocluster assembled film, the nanoclusters are pre-formed in an agglomeration/aggregation zone after sputtering and are subsequently made to deposit on a substrate by applying a pressure gradient across two chambers, namely, aggregation chamber and deposition chamber^23,35,36,41,42^. The microstructures, so produced, consist of isolated nanoclusters without grain boundaries that are separated by an average inter-cluster distance which depends on the deposition time. In this paper, we report our observations on nanocluster assembled films of a Ni_40_Pd_60_ alloy which is known to be metallic in the bulk form. However, in the nano-cluster assembled form, they display an activated behavior similar to the observations in^21–23,35^. By systematically varying the deposition time, we were able to make various nanocluster assemblies with varying average inter-cluster separations. We find that above a certain inter-cluster separation, the assemblies are three-dimensional in character that transform to two dimensions below that separation. In the three dimensional assemblies, we observe a change in the hopping transport from Efros-Shklovskii to Mott VRH as the average inter-cluster separation is varied. To our knowledge, this is the first direct observation of a systematic change in the transport mechanism, from the strongly localised transport of E-S kind to the weak transport of Mott variable range hopping kind, obtained by systematically varying the average inter-cluster separation.

## Ni_40_Pd_60_ Nanoclusters

**Figure 1:**
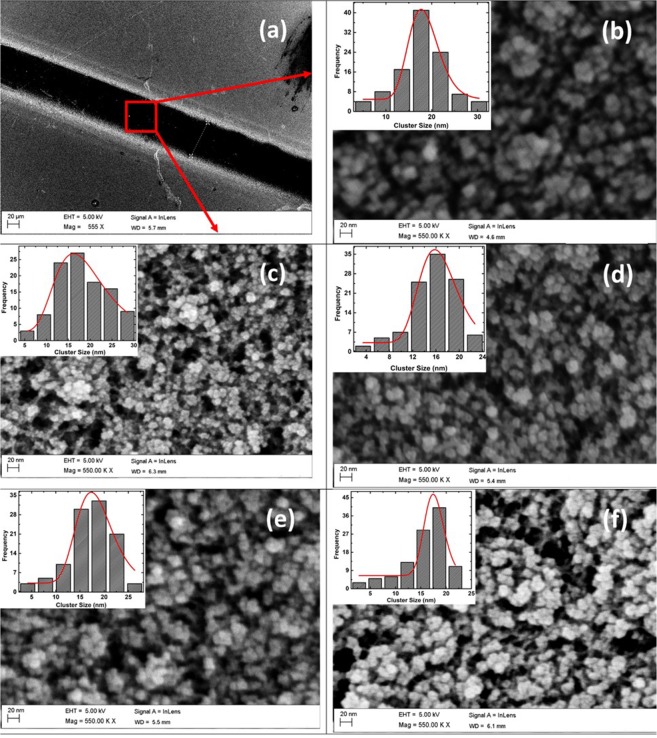
(**a**) FESEM image of the channel region where Ni_40_Pd_60_ nanoclusters where deposited. Red square and the arrows show the zoomed images of the deposited nanoclusters as seen in the main panels of (**b**–**f**) which show the FESEM images of nanocluster assemblies of Ni_40_Pd_60_ with deposition times of (**b**) 5 minutes (**c**) 7.5 minutes (**d**) 10 minutes (**e**) 15 minutes and (**f**) 20 minutes. Inset of each panel show the log-normal size distribution of the clusters.

Figure 1(b–f) show representative FESEM images of as-deposited Ni_40_Pd_60_ nanocluster assemblies corresponding to deposition times of 5, 7.5, 10, 15 and 20 minutes respectively. The insets to each figure show the calculated average cluster size obtained by fitting a log-normal distribution to the histograms obtained from the SEM images. The average inter cluster separations were determined using two softwares, namely, Image J and Scanning Probe Image Processor. Both the softwares give consistent results. From the histograms, it can be seen that the peak cluster size is about 16–17 nm for all deposition times, since, in the nano-cluster deposition system used, a quadrupole mass filter assembly doesn’t exist that can size select the clusters^36,41,42^. The average inter cluster separation obtained for 5, 7.5, 10, and 15 and 20 minutes of deposition are 14 nm, 11 nm, 6 nm, 2 nm and 0.7 nm respectively. For a deposition time of 20 minutes, the clusters are very close to each other and are on the verge of overlap. The corresponding assemblies have been named alphabetically for clarity. So, the 14 nm average inter-cluster separation nanocluster assembly is named A, 11 nm as B, 6 nm as C, 2 nm as D and 0.7 nm as E. It can be clearly seen that the average inter cluster separation of the as-prepared Ni_40_Pd_60_ nanocluster assemblies decreases with increasing the deposition time.

**Figure 2:**
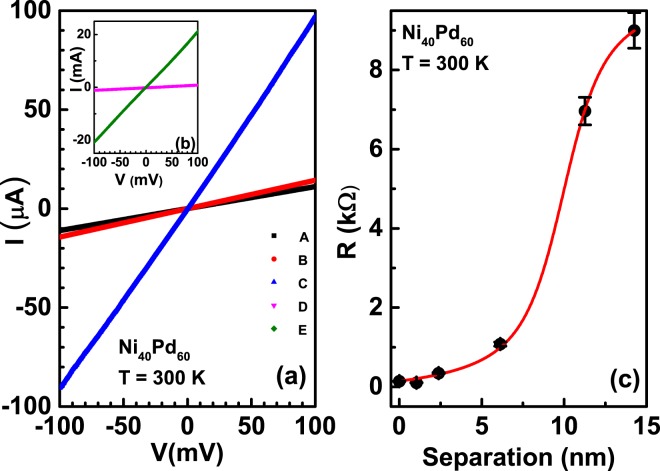
(**a**) I-V characteristics of Ni_40_Pd_60_ at 300 K for nano-cluster assembly with an average inter-cluster separation 14 nm (Assembly A, black colour), 11 nm (Assembly B, red colour and, 6 nm (Assembly C, blue colour). Inset (**b**) shows the I-V characteristics for average inter-cluster separation 2 nm (Assembly D, Pink colour) and 0.7 nm (Assembly E, Olive colour). (**c**) Filled circles show the room temperature resistance (R) variation with different inter-cluster separation. Red solid curve is the fit to the Langevins function. See text for details.

Figure 2(a) shows a representative I-V response of the assemblies A, B, C, D and E, corresponding to an average intercluster separation of 14 nm, 11 nm, 6 nm, 2 nm and 0.7 nm, respectively, measured at room temperature. It was found that the current-voltage response was linear in the voltage range −100 mV to 100 mV for all the assemblies. Main panel of Fig. 2(a) shows the I-V response for the assemblies A, B and C. It can be seen that the absolute value of the current is in the micro-ampere range. On the other hand, for the average inter-cluster separation of 2 nm and 0.7 nm, the absolute value of the current is three orders of magnitude larger at the mill-ampere range. The I-V characteristics of these assemblies (D and E), are hence, shown as an inset to Fig. 2(a). It can be seen that the resultant response of these assemblies are also linear in the range ±100 mV. Since the curves are Ohmic in the range ±100 mV, we were able to calculate the resistance of each film. Figure 2(b) shows the room temperature resistance of the Ni_40_Pd_60_ nanocluster alloy as a function of the inter-cluster separation. Black filled circles correspond to the calculated resistance. It can be seen that the resistance of the assembly decreases substantially as the inter-cluster separation decreases: for an assembly with an average intercluster separation of 14 nm, the room temperature resistance is ~**9 **kΩ while for an assembly with an inter-cluster separation of ~0.7 nm, the resistance is ~70 **Ω**, a three order of magnitude drop! This observation, then, suggests that the room-temperature resistance of the nanocluster assemblies of Ni_40_Pd_60_ alloy can be tuned by three orders of magnitude only by changing the inter-cluster separation. Red solid line in Fig. 2(b) is a fit to the Langevins function

4 $$R(x)={R}_{0}+c\ast (\coth (x-{x}_{c}))-\frac{1}{(x-{x}_{c})})$$

where R is the calculated value of resistance, x is the average inter-cluster separation, R_0_ is an offset, c a constant and x_*c*_ is the value at which the curvature changes from positive to negative. From the fit, x_*c*_ is ~10 nm.

It can be seen that the data is fitted very well with the Langevins function. Langevins function is known to describe the magnetisation data of non-interacting paramagnets very well. Here, the net magnetisation M of a paramagnet increases smoothly as a function of an external magnetic field H, wherein, the spins of the paramagnet keep aligning in the direction of the magnetic field until all the spins have aligned and a saturation is reached^43^. Similarly, in polymer physics, Langevins function is used to describe the polymerisation of monomers of some length L, wherein, each monomer gets extended by the application of an external force F smoothly until all the monomers have aligned in the direction of the force F and polymerisation achieved^44,45^. Since the Langevins function describes the resistance of the cluster assembled films varying with inter-cluster separation very well, by comparing this behaviour with that of paramagnets and polymers, we conjecture that increasing the inter-cluster separation has the same effect of increasing the resistance of each nano-cluster assembled film as that of increasing the magnetisation of paramagnets or that of extension length L of a monomer.

The variation of resistance with inter-cluster separation brings the question of the mechanism of charge transport in such cluster-assembled films. In order to estimate if tunnelling could be the main mechanism of charge transport, we did a rough calculation of the tunnelling probability of an electron in a metal with workfunction $\varphi$ (For Ni_40_Pd_60_, $\varphi$ ~ 5.5^46^), having some energy *E*_0_ and moving across a square barrier of width L^47^. The probability was found to be negligibly small for an inter-cluster separation of 14 nm and is appreciable only at inter-cluster separations below 1 nm. So, the main mechanism of charge transport in our nano-cluster assembled films seems to be due to hopping, from defect to defect, via electrons transiently localised at such sites. At low inter-cluster separation, where the hopping probability of the electrons is high, the resultant value of resistance is low. As the inter-cluster separation increases, the hopping probability decreases, which in turn, increases the resistance of the assemblies. In the limit of zero hopping (very large inter-cluster separation), the resistance value saturates to a very high value. In our nano-cluster assembled assemblies, this limit is reached around 14 nm.

## Change of Transport Mechanism and Dimension

**Figure 3:**
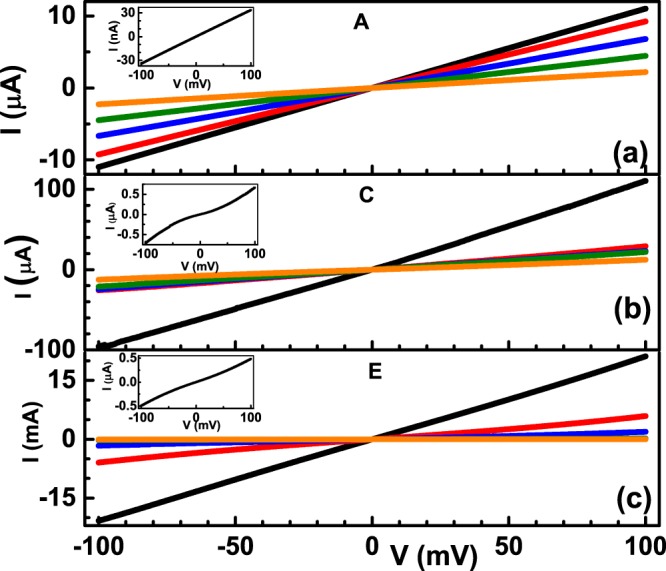
Temperature dependent I-V characteristics of nanocluster assemblies (**a**) A, (**b**) C and (**c**) E measured at temperatures of 300 K (black curve), 250 K (red curve), 200 K (blue curve), 200 K (olive curve) and 150 K (orange curve) at bias voltage range of −100 mV to 100 mV. The inset to each main panel shows the corresponding I-V at a lower temperature of 50 K.

Since the inter-cluster separation for the nano-cluster assembled films A to D varies from 14 nm to 2 nm, which is above the tunnelling transport limit as described above, the change of resistance by three orders of magnitude from assemblies A to D, implies differing levels of disorders, and consequently, differing transport mechanism in the different assemblies. In experiments of stress induced resistance change of monolayer MoS_2_ under conductive Atomic Force Microscope (AFM) configuration^48^, the resistance of MoS_2_ monolayers was found to change by three orders of magnitude. This change in the orders of magnitude was shown to arise due to the variation of the AFM tip-MoS_2_ layer distance which got modulated on the application of mechanical pressure from the AFM tip. The range of variation of the AFM tip-MoS_2_ layer distance was from 0.3 nm to 0.65 nm that brought about a change of resistance from ~3 MΩ to ~10^3^ MΩ. Similarly, Yu *et al*.^49^ studied the variation of the electrical resistance of carbon nanotubes dispersed in an insulating material as a function of the insulating film thickness. They found the resistance to change by 15 orders of magnitude when the thickness of the insulating film was changed from 0 to 1.8 nm! However, it is to be noted that the limit of tunnelling was reached at 1.8 nm in correspondence to the theoretical limit of tunnelling^50^. In order to understand the details of the transport properties of the nano-cluster assemblies with varying inter-cluster separation, we measured the temperature dependent resistivity of each assembly. Main panel of Fig. 3(a–c) shows the I-V curves of assemblies A, C and E respectively measured at temperatures of 300 K, 250 K, 200 K, 150 K and 100 K. It was found that all the assemblies exhibited a linear I-V till a temperature of about 50 K. Below this temperature, some assemblies exhibited a non-linear I-V. The inset of Fig. 3 shows the I-V of assemblies A, C and E at 30 K. It is to be noted that for the assembly A, the I-V is linear even at 30 K but for C and E, non-linearity sets in at 30 K. The value of resistance at each temperature was obtained from the Ohmic I-V from 50 K to 300 K.

A large amount of literature on granular metal films^24,28–32^ has established the fact that a combination of linear as well as non-linear I-V characteristic describes the films, depending on the temperature range being investigated. At higher temperatures (≥100 K), the curves are linear while below 100 K, non-linearity sets in. Similarly, in chemically reduced graphene oxide sheets with varying amounts of sp^2^ fractions, a similar combination of linear as well as non-linear I-V characteristics was obtained depending on the temperatures at which the measurements were done^19^. Above 150 K, the I-V curves were found to be linear while below 150 K, non-linearity was found to set-in. Very different transport mechanisms describe the two systems: for granular metal films, charge transport happens via tunnelling while for the graphene oxide sheets, strong disorder dictates an Efros-Shklovskii hopping mechanism of charge transport. The non-linear I-V characteristics in the granular metal films are understood to arise due to electron tunneling from one grain to the other resulting in a transition between two different states of charge in the pair of grains^28^. To our knowledge, a similar explanation for non-linear I-V characteristics at low temperatures in systems exhibiting Efros-Shklovskii transport does not exist. It is hoped that the observations from our experiments will stimulate further theoretical works in order to explain the temperature dependent I-V characteristics in nanocluster assembled films.

**Figure 4:**
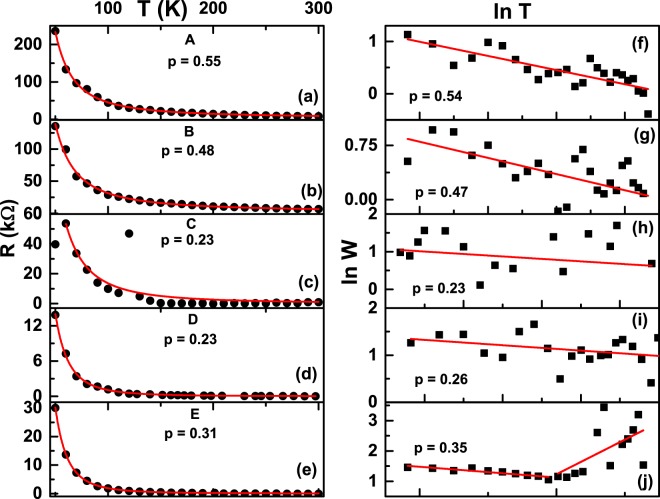
Resistance (R) vs. temperature (T) variation of nanocluster assemblies (**a**) A, (**b**) B, (**c**) C, (**d**) D and (**e**) E. Filled circles are data points while red curves in (**a**–**e**) are exponential fits. See text for details. Reduced activation energy (W) vs. temperature T plotted on a log-log scale for nanocluster assemblies (**f**) A, (**g**) B, (**h**) C, (**i**) D and (**j**) E. Red solid line in (**f**–**j**) are least square fits to a straight line.

Figure 4(a–e) plot the temperature dependence of resistance for devices A through E for temperatures where the I-V in Fig. 3 was linear, namely, 50 K to 300 K. Black filled circles in each curve correspond to the data points. It can be clearly seen that all the curves exhibit an activated behavior, in complete contrast, to what is expected for Ni_40_Pd_60_ alloy which is a bulk metal. By studying the evolution of the density of states in ultrathin films of Be (which is a metal in bulk form) of varying resistances, Butko *et al*.^21^ found that Coulomb gap mediates the insulating behavior. In order to check if the observed activated behavior may have a similar reason, we fitted the temperature dependent resistance data of Fig. 3 to an equation of the following form:

5 $$R={R}_{0}{\exp }{({T}_{0}/T)}^{p}$$

where *R*_0_ is the zero temperature value of the resistance, *T*_0_ is a constant and p is the hopping exponent whose value determines the kind of hopping mechanism at work. It can be seen that all the curves fit the data very well. The fit to the data of nanocluster assembly C is not so good. However, from the I-V plot of Fig. 2(b), it is clear that the I-V data is quite good. So, the anomalous R-T data of Fig. 4(c) may represent anomalies of the physical system itself.

From the fit, the hopping exponent was obtained as 0.55, 0.48, 0.23, 0.23 and 0.31 for assemblies A, B, C, D and E respectively. The differing values of the exponents are indicative of different transport mechanism in the different nano-cluster assemblies. To check the self-consistency of the obtained hopping exponents, we replotted the data of Fig. 4(a–e) by calculating the logarithmic derivative^51–53^:

6 $$W=-\,\frac{\partial \,{ln}\,R(T)}{\partial \,{ln}(T)}=p{(\frac{{T}_{0}}{T})}^{p}$$

The hopping exponent p is then obtained from the slope of the lnW vs. lnT plot since lnW = A − p * lnT.

Figure 4(f–j) show lnW-lnT plots for assemblies A, B, C, D and E respectively. In order to obtain accurate values of the hopping exponent, a least square fit to the data was done. Black filled squares are the data points while the red curve is the straight line fit. It can be seen that the straight line fits the data very well. The values of p obtained from the fits are 0.54, 0.47, 0.23, 0.26 and 0.35 for assemblies A, B, C, D and E respectively, in excellent agreement to the values obtained from the exponential fit. From Fig. 4(j), it can also be observed that a straight line fit with a negative slope of 0.35 is possible only till T ~150 K. Above this temperature, the slope of the curve changes from negative to positive, indicating a possible cross-over to a metal-like state. The small values of resistance of assembly E in the range of 470 Ω (at 150 K) to 70 Ω (at 300 K) supports this conjecture. It is to be noted that no corresponding change in slope of R-T is observed in Fig. 4(e). A possible reason for this could be that the function W is a derivative function of R(T), so it can capture slope changes of a weakly changing function (R (T)) much better than the function itself. Hence, the slope change of lnW-lnT data may indicate a very weak cross-over from an insulating to a metal-like change of transport, not visible in the corresponding R-T data.

**Figure 5:**
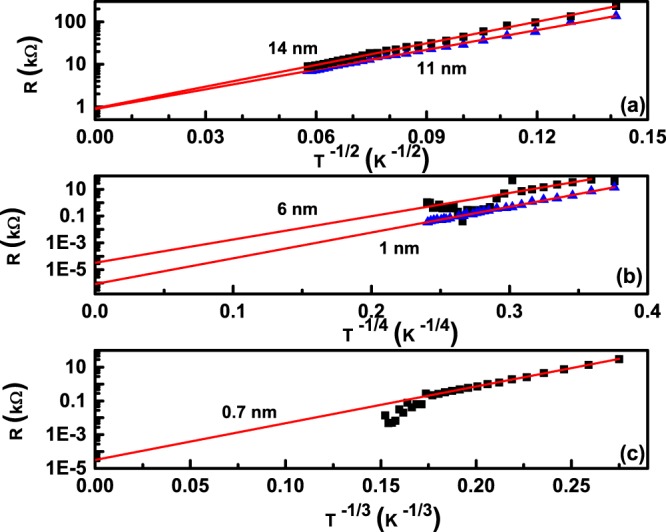
Semi-logarithmic plots of (**a**) R vs. T^−1/2^ for nanocluster assemblies A and B (**b**) R vs. T^−1/4^ for nanocluster assemblies C and D and (**c**) R vs. T^−1/3^ for nanocluster assembly E. Symbols are data points while red solid lines are straight line fits.

If R indeed varies as T^−1/2^ for assemblies A and B; as T^−1/4^ for assemblies C and D; as T^−1/3^ for assembly E, then a plot of lnR with T^−1/2^, T^−1/4^ and T^−1/3^ should be a straight line. In order to confirm this, as well as, estimate the values of R_0_ and T_0_, we made a plot of ln R *vs*. T^−1/2^, T^−1/4^ and T^−1/3^. Figure 5(a) plots lnR vs. T^−1/2^ for assemblies A and B, Fig. 5(b) plots lnR vs. T^−1/4^ for assemblies C and D and Fig. 5(c) is the plot of ln R vs. T^−1/3^ for assembly E. Symbols represent the data points while the red line is a straight line fit to the data points. It can be clearly seen that all the curves fit perfectly to a straight line, thus reiterating the values of exponents 1/2 for assemblies A and B, 1/4 for assemblies C and D and 1/3 for assembly E. It can also be observed that the data corresponding to 6 nm average inter-cluster separation assembly is a bit noisy, as discussed above, while the data corresponding to 0.7 nm average inter-cluster separation assembly deviates from linearity below T^−1/3^ = 0.174 which corresponds to T = 150 K, in conformity to the observations from Fig. 4(j).

It is clear from the observations of Figs. 4 and 5 that the hopping exponent is 1/2 for nano-cluster assemblies A and B suggesting an Efros-Shklovskii (E-S)^10,11^ mechanism of transport in them. The high value of resistances in A and B also supports this scenario^21^. This observation, then, suggests that the nano-cluster assembled assemblies A and B, having an average inter-cluster separation of 14 nm and 11 nm, are disordered enough to support an E-S mechanism of charge transport. Such a transport is expected in systems that are so strongly localized that the density of states N(E_*F*_) at the Fermi-level are not flat but vary linearly with energy, eventually reaching zero values at the Fermi level due to long-range electron interactions between localized states. A strong localization is expected in nano-cluster assembled assemblies A and B which have a large inter-cluster separation.

From the intercept of the fits, the zero temperature resistance R_0_ was obtained for the assemblies corresponding to different inter-cluster separation values. R_0_ was seen to collapse to 925 ohms for assemblies A and B (within 40 ohms). For assemblies C, D and E, R_0_ was found to be 32 mΩ, 0.8 mΩ and 32 mΩ respectively. The characteristic temperature T_0_ was obtained from the slope of the fits and the values are 288 K, 240 K, 91248 K, 136765 K and 10319 K for nanocluster assembly A, B, C, D and E respectively. The high values of the characteristic temperature T_0_ are similar to what was obtained in graphene oxide sheets^19^. It is well-known that for Efros-Shklovskii mechanism of charge transport, T_0_ can be expressed as:

7 $${T}_{0}={T}_{ES}=\frac{2.8{e}^{2}}{4\pi \epsilon {\epsilon }_{0}{k}_{B}\xi }$$

where $\epsilon$ is the dielectric constant of the material and $\xi$ is the localization length.

Since Ni_40_Pd_60_ is a metal in the bulk form, its bulk dielectric constant values are not known. Since the assemblies A and B are shown to follow Efros-Shklovskii mechanism of charge transport very well where the charge carriers are known to be strongly localised at a defect site, it is reasonable to assume a small value of the localization length $\xi$ as ~1 nm^21^. Using this value in equation 7, we get the dielectric constant $\epsilon$ as 162 and 195 for nanocluster assembly A and B respectively.

As the inter-cluster separation is reduced to 6 nm and 1 nm in assemblies C and D respectively, the hopping exponent was found to be 0.23. This number is strikingly close to the exponent 1/4, expected for a Mott variable range hopping mechanism. This mechanism is expected to set-in for disorder strengths of such a magnitude that the density of states N(E_*F*_) at the Fermi level is constant^4–6^. For a constant N(E_*F*_) at the Fermi level, Mott showed that p in equation 1 is given by equation 3:

$$p=\frac{1}{D+1}$$

where D is the dimenionality of the system.

For p = 1/4, D turns out to be 3. So, the above analysis suggests that the dimensionality of the nanocluster assemblies A, B, C and D is 3. This is not surprising in view of the fact that the metal cores of many Pd nanoclusters are known to exhibit structural characteristics similar to that of the parent metal themselves^54–57^. For example, the palladium atoms in Pd_10_(µ_3_-Co)_4_(µ_2_-Co)_8_(p-Bu^*n*^_3_)_6_ form a 10-vertex polyhedron^54^, those in [Pd_7_(Co)_7_(PMe_3_)_7_] form an octahedron^55^ while the Pd atoms in Pd_38_(Co)_28_(PEt_3_)_12_ arrange themselves in a distorted cubic arrangement^57^. Since the dimensionality is 3 for all the nano-cluster assemblies A, B, C and D, it seems that the three dimensional character of the assembly is retained till an inter-cluster separation of 1 nm.

The direct observation of a change in hopping mechanism from E-S kind to that of Mott variable range kind by systematically changing the inter-cluster separation is very interesting and not previously observed, to our knowledge. The previous observations of a similar change in temperature dependence from T^1/2^ to T^1/4^ or from T^1/2^ to T^1/3^ were in compensated n-type InP semiconductor and GaAs/Al_0.3_Ga_0.7_As heterostructures respectively, where the said transition was obtained by changing the magnetic field^12,17^: below a threshold field, the conductivity varied as T^1/4^ (T^1/3^), while above the critical field, it varied as T^1/2^. In the compensated n-type semiconductor, for fields below the critical field, the sample was on the insulating side of the metal-insulator (M-I) transition and the variable range hopping mechanism was ascribed to that of Mott kind while far from the M-I transition, the hopping conduction happened via E-S hopping mechanism due to the opening up of a Coulomb gap. In our case, the analysis from Figs 4 and 5 clearly indicate that for inter-cluster separations of 14 nm and 11 nm, the hopping mechanism is of E-S kind while for the inter-cluster separations of 6 nm and 1 nm, the transport happens via the Mott variable range hopping mechanism. It is known that the width of the Coulomb gap E is given by^6^:

8 $${\rm{\Delta }}E=\frac{1}{4\pi a{\xi }^{2}N({E}_{F})}$$

where $\xi$ is the localization length and N(E_*F*_) is the density of states at the Fermi level. When the inter-cluster separation is large, the localization length is small as is the case for the nanocluster assemblies A and B. As the inter-cluster separation decreases, the localization length increases and Δ*E* tends to zero for nanocluster assemblies C and D. For an overlapping cluster, $\xi \to \infty$ and Δ*E *$\to$ 0. The decrease in the Coulomb gap results in the closing up of the gap at the Fermi level and a constant density of states, thus initiating a Mott transition from a E-S mechanism of transport, as observed.

Finally, when the inter-cluster separation has reduced quite a lot (~0.7 nm), the electronic wavefunctions overlap sufficiently. This results in the formation of a continuous film like structure in the nanocluster assembly with a reduced dimensionality of two. The hopping exponent p, should then change to 1/3 from 1/4. This is exactly what is obtained from the temperature dependence of the nano-cluster assembly E, as observed in Fig. 4 where the hopping exponent was found to be ~0.33 (c.f. Fig. 4(e,j)), while ln R was found to vary linearly with T^−1/3^ in Fig. 5(c). It has been shown above that for an inter-cluster separation of 0.7 nm, charge transfer could also happen via tunnelling. In this mechanism of charge transfer, R is expected to vary as T^−1^ ^32^. However, if transport happens via Mott variable range hopping, then R should vary as T^−1/3^ ^34^. From Figs 4(e,j) and 5(c), it is clear that R varies as T^−1/3^, suggesting that the main mechanism of charge transport in the assembly E is that of Mott-variable range hopping rather than tunnelling.

## Conclusions and Outlook

In conclusion, we have demonstrated that nanoclusters offer a novel playground for studying the important problem of charge transport in a physical system as the system is slowly built up by making a nano-cluster comprising some number of atoms whose inter-cluster separation is then varied. When the inter-cluster separation is large, of the order of 10–14 nm, there ia a strong charge localisation due to the opening up of a Coulomb gap at the Fermi level. The resultant charge transport, is then, of Efros-Shklovskii hopping. The spatial dimension of such assemblies is found to be three. As the inter-cluster separation is decreased below 10 nm, the charge transport mechanism changes from Efros-Shklovskii hopping to a Mott variable range hopping due to the closing up of the gap at the Fermi surface. To our knowledge, this is the first direct observation of a change in transport mechanism from Efros-Shklovskii hopping to a Mott variable range hopping obtained by directly varying the average inter-cluster separation. The previous observations of a similar change in transport mechanism has been indirect, via either a change of concentration or that of an external magnetic field. Finally, when the average inter-cluster separation has reduced below 0.7 nm, the nano-cluster assembly takes the form of a quasi-continuous film in which the dimensionality was found to reduce to two as inferred from the Mott variable range hopping exponent change from 1/4 to 1/3. In the quasi-continuous film, we also observed a temperature dependent insulator to weak metal-like transition. Thus, by systematically varying the average inter-cluster separation from large (resulting in isolated three dimensional assemblies) to small (leading to two dimensional quasi-continuous films), charge transport of one extreme (strong localisation of Efros-Shklovskii) to the other extreme (weakly metallic) was obtained by the intermediate weak transport (Mott-variable range hopping), thus offering nano-clusters as an excellent model system to study charge transport in a system of varying number, size and inter-cluster separation.

## Materials and Methods

Ni_40_Pd_60_ alloy nanoclusters were deposited in the channel region of a pre-fabricated bottom gate structure. The substrate was a commercially obtained Si wafer on which a Gate oxide (SiO_2_) layer of thickness 100 nm was used in order to make the layer insulating. Prior to the deposition of Ni_40_Pd_60_ nanoclusters in the channel region, a self-assembled monolayer (SAM) of Trychlorocyclohexyle Silane (TCCHS) was made on the SiO_2_ gate oxide by spin coating in the anhydrous condition of a Ar glove box (Model MB20 G-MBraun). The O_2_ and H_2_O level was maintained at less than 0.1 ppm during the experiment. For making the electrical contacts, gold (Au) electrodes of thickness 100 nm were made by RF sputtering of Au metal on the SAM. The sputtering deposition was carried out at a working pressure of 4.5 × 10^−3^ torr and a magnetron power of 60 Watts. The channel between the electrodes was formed by using a 0.17 mm diameter gold wire as shadow mask. The channel length was 100 micrometer and channel width was 2 mm.

Nanocluster assemblies of Ni_40_Pd_60_ alloy were deposited on the channel region made above as shown in Fig. 1(a). The deposition of Ni_40_Pd_60_ nanoclusters was done using a Nanocluster Deposition System (Model Nanodep60 from Oxford Applied Research, UK). In this system, atoms of the target material get sputtered by a dc magnetron and allowed to move in an aggregation chamber along with a carrier Argon gas of 99.99 percentage purity. Nanoclusters are formed in this aggregation chamber and are forced to go through an aperture into a deposition chamber which is maintained at a slightly lower pressure than the aggregation chamber by differential pumping mechanism. The distance between the substrate and apertures was about 55 cm. The base pressure achieved in the system before the start of deposition was 4.7 × 10^−7^ mbar and a working pressure of 5.5 × 10^−5^ mbar was obtained during the aggregation gas flow whose flow rate was maintained at 100 sccm. The current in the DC magnetron was held constant at 0.2A and the power level was about 75 Watts for all the depositions of the present system. The nanoclusters were deposited for different durations of exposure times ranging from 5 minutes to 20 minutes which resulted in different degree of overlap of clusters, and hence, different average inter cluster separations. Thus, the deposition time was the control parameter for making Ni_40_Pd_60_ cluster assemblies with different average inter cluster separation.

The micro structural characterization of the assemblies was done using a Field Emission Scanning Electron Microscope (Model Ultra 55 from Carl Zeiss, Germany). The operating voltage of FESEM used was 5 kV with a working distance of 4–6 mm. Current versus voltage (I-V) measurements were carried out using Semiconductor device analyser (Agilent B1500 A) equipped with three source measure units (SMU). Two of the SMUs were high resolution units whereas one of them was a high power unit. The temperature of the nanocluster assembled film was varied in the range of 5 K–300 K using a Cryogenic Probe Station (Model CRX-4K from Lake Shore Cryotronics). The three probes (tips of Be-Cu or W) were connected to the Source, Drain, and Gate terminals of the device. The temperature inside the probe station was controlled by two temperature controllers (Lakeshore Model 336), one of which was near the sample and the other at the cold head.
